# LightFD: Real-Time Fault Diagnosis with Edge Intelligence for Power Transformers

**DOI:** 10.3390/s22145296

**Published:** 2022-07-15

**Authors:** Xinhua Fu, Kejun Yang, Min Liu, Tianzhang Xing, Chase Wu

**Affiliations:** 1 School of Information Science and Technology, Northwest University, Xi’an 710100, China; fuxinhua@stumail.nwu.edu.cn; 2 Anhui Nanrui Jiyuan Electricity Grid Technical Co., Ltd., Hefei 230088, China; yangkejun@sgepri.sgcc.com.cn; 3 Anhui Zhongke Haoyin Intelligent Technology Co., Ltd., Hefei 230000, China; zkhyxuquan@outlook.com; 4 Department of Computer Science, New Jersey Institute of Technology, Newark, NJ 07102, USA

**Keywords:** power transformer, Mel Frequency Cepstrum Coefficient (MFCC), sound signal, fault diagnosis, spectrogram

## Abstract

Power fault monitoring based on acoustic waves has gained a great deal of attention in industry. Existing methods for fault diagnosis typically collect sound signals on site and transmit them to a back-end server for analysis, which may fail to provide a real-time response due to transmission packet loss and latency. However, the limited computing power of edge devices and the existing methods for feature extraction pose a significant challenge to performing diagnosis on the edge. In this paper, we propose a fast Lightweight Fault Diagnosis method for power transformers, referred to as LightFD, which integrates several technical components. Firstly, before feature extraction, we design an asymmetric Hamming-cosine window function to reduce signal spectrum leakage and ensure data integrity. Secondly, we design a multidimensional spatio-temporal feature extraction method to extract acoustic features. Finally, we design a parallel dual-layer, dual-channel lightweight neural network to realize the classification of different fault types on edge devices with limited computing power. Extensive simulation and experimental results show that the diagnostic precision and recall of LightFD reach 94.64% and 95.33%, which represent an improvement of 4% and 1.6% over the traditional SVM method, respectively.

## 1. Introduction

In recent decades, the rapid growth of the global energy industry along with economic development and continuous social progress has led to an increasing demand for electric energy [1]. Among the power equipment, power transformers, as the primary source of changing voltage, are one of the most important components to maintain the robust operation of power systems. They play an indispensable role in the power transmission and distribution systems [2]. Transformers are subject to various types of failures throughout their lifecycle, including production, installation, maintenance, and prolonged operation. Once a fault occurs, it may not only cause severe damage to the equipment itself, but also pose a significant threat to the safety of people and the reliability of power supply [3,4]. Therefore, it is an important problem to detect faults and identify their types with high accuracy in a timely manner. Therefore, appropriate measures can be taken to mitigate the negative effects.

Existing methods for fault diagnosis largely rely on special equipment such as contact sensors. Such methods increase the cost of fault diagnosis and, more importantly, may be affected by high voltage and strong electromagnetic fields and other complex working environments, hence interfering with the normal operation of the system. The inner winding and iron core of the transformer realize the important function of electromagnetic exchange. The internal vibration of the operating transformer, including the periodic vibration caused by the magnetostrictive effect of the core silicon steel sheet and the winding vibration generated by the electric potential, radiates different amplitudes and frequencies of the vibration signal to the surroundings [5,6]. In particular, in high-voltage and strong electromagnetic environments, various faults may occur and produce different sounds. Fault diagnosis through sound signals presents a promising solution with multifold advantages. First of all, it supports non-contact installation and facilitates signal acquisition with small and simple equipment. Furthermore, acoustic signals do not generate electromagnetic fields and do not affect the normal operation of the equipment. In fact, acoustic recognition has been widely used in sound verification [7,8], healthcare [9,10,11], fault diagnosis [12,13,14], and many other applications.

Acoustic signal-based fault diagnosis typically consists of two steps, i.e., sound feature extraction and fault type classification. For sound feature extraction, a frequency domain transformation is typically required to extract feature parameters for recognition. Considering the similarity between the sound signals of power transformers and the human voice, commonly used feature parameters include the Mel Frequency Cepstrum Coefficient (MFCC), Linear Predictive Cepstrum Coefficient (LPCC) [15,16], Cochlear Filter Cepstral Coefficient (CFCC) [17], and perceptual linear prediction [18]. Sound recognition technology has been widely used to identify speakers. It creates a feature vector library by extracting the feature vectors of sound signals of different speakers and then compares the similarity of the feature vectors to determine the speaker’s identity. Since the sound signal produced by a working transformer is somewhat similar to the human voice, it is possible to effectively extract the characteristics of transformer noise using the typical parameters describing the human voice. Transformer sound signals in operation contain abundant equipment information and are closely related to the transformer structure and operation state [19]. MFCC effectively reveals the time domain and frequency domain features of fault sound signals, so it is applied to the feature extraction of transformer sound signals in this paper. However, MFCC contains the static information of sound signals, but during a transformer fault, the relevant parameters constantly change towards the fault state. Therefore, we also need to extract the dynamic features of sound signals.

An acoustic classifier is a critical component for sound recognition. In recent years, many machine learning algorithms such as Artificial Neural Networks (ANNs), Support Vector Machines (SVMs) [20,21], and decision trees have been studied in the literature as promising solutions to transformer fault detection. Among them, the combination of signal processing techniques and support vector machines has attracted increasing attention from researchers due to its ability to tackle challenging problems such as “dimensional catastrophe”, “overfitting”, and local minima. Although ANNs have strong capabilities of self-learning and parallel processing, they converge rather slowly and sometimes may fall into local optima [22]. In contrast, Deep-Learning (DL)-based algorithms enable automatic representation and abstract feature extraction due to fast iterations and GPU-based parallel implementation.

Deep learning has been widely used in human–computer interaction classification. At present, most of the existing work focuses on deep learning frameworks and uses a general network for recognition and classification. The sound-based fault detection methods can be divided into two categories according to the data processing workflow in the system. The first category collects signals through sound sensors and transmits them to the server [23,24]. This process may suffer from packet loss and transmission delay as data acquisition and transfer are subject to wild fluctuations in complex operating environments. This poses a significant challenge for the accuracy and timeliness of fault diagnosis. The second category is in situ diagnosis using a lightweight system deployed on low-end equipment to avoid data transmission. Note that the complexity of a neural network largely determines the performance of the model. As the network complexity increases with more parameters, the required computational effort and the demand for training samples also increase. Existing techniques for parameter compression usually degrade the performance of the model. Since low-end edge devices have limited computing power, there is a greater need to design a lightweight system using a simple model for fast in situ diagnosis on edge devices.

In this paper, we propose to develop a real-time fault diagnosis system with edge devices for power transformers, referred to as LightFD, for fast fault detection and accurate type identification. LightFD integrates a Hamming-cosine window to reduce spectral leakage, a new method for multidimensional spatio-temporal feature extraction. A parallel dual-layer, dual-channel lightweight neural network would address the issue of the limited computing power of edge devices. The design of this system faces the following challenges.

**How do we design a suitable window function?** Before performing feature extraction of the sound signal, we need to decide an appropriate window function to multiply the framing signal with the original sound signal in the time domain. This window function has a great impact on the performance of transformer fault diagnosis. Ideally, the window spectrum should have a narrow main lobe and a side lobe with a fast decay. We design a Hamming-cosine window for each frame for further processing.

**How do we fully extract the dynamic information of fault sound signals?** In general, transformers progressively malfunction over a certain period. Therefore, the relevant parameters are constantly changing towards the fault state. Dynamic features also contain rich transformer state information, which can be used to improve the accuracy of transformer fault diagnosis. It is important to extract and filter dynamic features from different perspectives to improve the quality of input data for the subsequent lightweight neural network. To address this challenge, we designed a multidimensional spatio-temporal feature extraction method. Relative-MFCC (the dynamic feature of sound signals) is designed on temporal feature extraction to fully extract dynamic features.

**How do we design a neural network with less complexity?** The complexity of a neural network can directly affect the accuracy of the model. Lightweight structures (through various techniques such as simplifying the hierarchy, compressing the number of parameters, etc.) usually degrade the accuracy of the model. Due to the limited processing power and storage space of edge devices, the neural network deployed on them must be lightweight. To reduce the network complexity, we design a parallel dual-channel network to extract spatial features and temporal features. We construct lightweight point-state convolutional units as the main components of the dual-layer, dual-channel network to further reduce the complexity of the network. In addition, due to the redundancy of neural networks, we design a linear variation method to extend the number of features.

We deploy the proposed LightFD system on a Raspberry Pi 4B device and conduct experiments for performance evaluation. We use non-contact sensors to collect sound signals emitted by the transformer, solving the interference problem caused by directly attaching sensors to the exterior surface of the transformer being monitored. The Raspberry Pi device receives sound signals from non-contact sensors and then performs fault diagnosis of the transformer. Extensive results show that LightFD achieves a recognition precision and recall of up to 94.64 and 95.33%, respectively, in a relatively short time, which represent an improvement of 4% and 1.6% compared with the traditional SVM method. The contributions of our work are summarized as follows:

- Our proposed system identifies six types of faults: large load start-up (Large load start-up is a special condition in transformer operation, and the frequent occurrence of this condition will make transformer faults increase. Therefore, we include large load start-up as a diagnostic object.), severe internal short circuit, internal breakdown short circuit, poorly grounded iron core, loose silicon steel or coil, and high voltage. When the system diagnoses a fault, it can generate early warnings about various states of the transformer in a timely manner.
- We design a multidimensional spatio-temporal feature extraction method to obtain and fuse the dynamic features of faulty sound signals from different angles in multiple dimensions.
- We design a lightweight network for low-end edge equipment to enable quick identification of transformer faults.

The rest of the paper is organized as follows. In Section 2, we review the related work. In Section 3, we present the system architecture and detail the design of components techniques. In Section 4 and Section 5, we present the implementation details and the evaluation results. We conclude our work in Section 6.

## 2. Related Work

### 2.1. Conventional Approaches

The emergence of acoustic recognition has facilitated the development of transformer fault detection, which can be monitored by a variety of common methods of sound diagnostics for transformer fault types. The commonly used methods are subjective evaluation and estimation, acoustical intensity analysis, fast Fourier analysis, wavelet analysis, Empirical Mode Decomposition (EMD) [25], etc., which can identify some types of transformer faults. For example, the subjective evaluation and estimation method refers to the use of a person’s own hearing to determine the type of equipment fault, which is somewhat subjective. The sound intensity analysis method uses the mutual spectrum method to measure the sound intensity of the equipment by collecting signals from two sensors simultaneously. Kendig et al. [26] proposed a new method for sound diagnosis by the “sound intensity measurement method”. This method can detect the operating condition of transformers in a certain background environment, but it is difficult to classify the fault type. Sykora et al. [25] proposed to use the EMD method to decompose the transformer sound signal and obtain the marginal spectrum by the Hilbert transform, which can compare and contrast the acoustical signal of the transformer in normal and overload conditions. Considering the similarity between the acoustic signal of the power transformer and human sound and the good anti-noise ability of the human auditory system, it is increasingly important to detect abnormal states of power transformers using acoustic signals. In [27], the MFCC component of a dry transformer acoustic signal was calculated and optimized. According to the optimized MFCC characteristic parameters, the transformer core loosening was identified by the Vector Quantization (VQ) algorithm.

In the past few decades, many hand-crafted features and conventional machine learning approaches have been proposed. The traditional machine-learning-based approaches for sound signal classification are shallow models with manually constructed features as the input. The most commonly used algorithms in classification tasks are Logistic Regression (LR) [28], Support Vector Machine (SVM) [20,21], Random Forest (RF) [29,30], Bayesian Network (BN) [31], and K-Nearest Neighbors (KNN) [32]. In addition, researchers often use hybrid techniques or model integration to enhance the overall model performance.

The most common acoustic feature inputs to these models are MFCC and LPCC-related features. The input features for these models can be found in [33,34]. These features are considered to be suitable indicators of short-term and long-term changes in sound signals.

### 2.2. Deep Learning Approaches

In recent years, the Convolution Neural Network (CNN) has been applied to fault diagnosis. Compared with traditional manual engineering features, the CNN can automatically extract effective features from input data through multi-level convolution and pooling operations, which is often more efficient than manually selected features. In view of the good performance of the CNN, the model is introduced in one-dimensional signal fields such as speech, voice recognition, and fault diagnosis [35,36]. Zhang et al. [37] presented a novel transformer fault diagnosis method using an Internet of Things (IoT)-based monitoring system and ensemble machine learning (EML). This kind of method is more affected by the network environment and is prone to packet loss and delay. In addition, the Recursive Neural Network (RNN) has also achieved great success due to the timing of signals. Do et al. [38] proposed a CNN to classify six kinds of discharge defects in power transformers. Dang et al. [39] proposed a fault diagnosis method based on the GFCC sound pattern spectrum and the CNN in order to better identify the normal state of the power transformer by sound signals.

Transformers usually fail gradually, not suddenly. Accordingly, the related parameters change continuously towards the fault state. Time analysis methods can be used to model the sequential dependence of state parameters with time. Tian and Zuo [40] developed a gearbox health state prediction method based on an Extended Recursive Neural Network (ERNN). Experimental results showed that the ERNN method can effectively evaluate the health status of the gearbox and play a role in fault prediction. Kong et al. [41] proposed a framework based on the LSTM RNN to solve the short-term load forecasting problem of an individual electric customer. The Long Short-Term Memory (LSTM) network [42,43], as an improved structure of the RNN, alleviates the problems of gradient dissipation and explosion in the long-term modeling process of the RNN to a certain extent and has attracted the attention of academic circles.

## 3. System Overview

**Figure 1:**
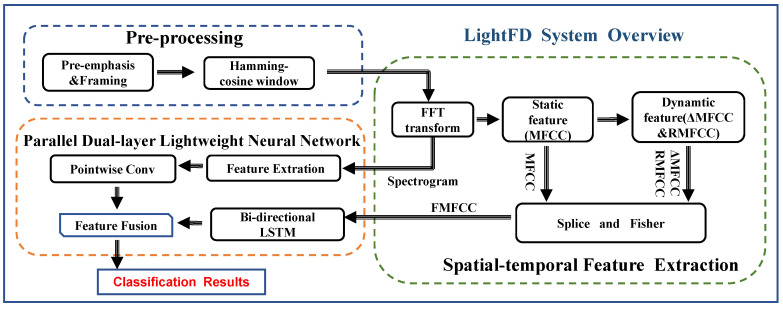
System overview.

LightFD is a real-time power transformer fault diagnosis system, whose architecture diagram is shown in Figure 1. It consists of three components: signal pre-processing, spatio-temporal feature extraction, and parallel dual-layer, dual-channel lightweight neural-network-based classification. Pre-processing consists of pre-emphasis, framing, and windowing. We used acoustic features obtained from short-term Fourier transformation as acoustic spectrograms and Filtered-MFCC (FMFCC) by splicing and filtering static features with dynamic features. Spectrograms and FMFCC are visual features and time-dependent features, respectively. Finally, we designed a parallel dual-layer, dual-channel lightweight neural network for edge devices to guarantee the speed and accuracy of diagnosis.

Transformer sound signals have time-varying, non-linear characteristics due to the influence of load current and some uncertain interference factors. To obtain relatively smooth transformer acoustic signals, we pre-emphasized the signals to enhance the effect of high-frequency, frame pre-emphasized sound signals by dividing them into short-time stable frame segments and designed a hybrid Hamming-cosine window to further process each frame to reduce spectral leakage [44].

The sound signal generated during transformer operation contains rich information, which can reflect the working status and fault condition to a certain extent. In this component, we implement MFCC-based time sequence feature extraction and spectrogram-based visual feature extraction of sound signals, respectively. The obtained MFCC reflects only the static feature of sound signals. To reflect and extract the dynamic features of fault sound signals, we considered utilizing ΔMFCC, which is the first-order differential MFCC. In addition, we designed a Relative-MFCC (RMFCC) that reflects the dynamic trend of sound signals. We observed that the direct superposition of the above features (36 dimensions) increases the computational effort and some redundant features might be present. Therefore, we used the Fisher ratio to filter the extracted static and dynamic features to improve diagnosis performance. We refer to the Filtered-MFCC features as FMFCC.

Deep learning networks involve the heavy computational overhead of convolutional operations and result in the redundancy of extracted features. To address the problem of the limited processing power of edge devices and to save transmission time in the process, we designed a parallel Dual-layer, Dual-channel Lightweight neural network (LightDD) to learn the visual and time-dependent features of transformer faults. In LightDD, we extract the features of each channel (note that this channel is not the channel in the dual-channel network), perform a linear transformation, and finally, use the point convolution method for multi-channel fusion. In this network, BiLSTM uses acoustic Filtered-MFCC (FMFCC) feature sequences as the input, while another channel uses the spectrogram as the input. Finally, the outputs of the parallel neural network are fully connected for feature fusion. Compared with traditional networks, our lightweight neural network has the advantages of fewer parameters and less computation.

## 4. Proposed Fault Diagnosis Method

In this section, we present the design of our proposed method with three major components: (i) pre-process sound signals of transformer faults; (ii) extract the features of sound signals and obtain the spatial visual features and time-dependent features of sound signals, respectively; (iii) design a lightweight neural network to classify transformer fault types.

### 4.1. Pre-Processing

Sound signals’ pre-processing is the basis of the entire fault diagnosis system, including signal pre-emphasis, framing, and windowing.

In fact, the power spectrum of speech, music, etc., decreases with increasing frequency, and most of its energy is concentrated in the low-frequency range. Therefore, the generated signal amplitude is caused by the low-frequency components of the signal because there is a significant attenuation in the high-frequency components of the signal [45]. Pre-emphasis is a type of processing that compensates for the high-frequency components of the original signal. The pre-emphasis filter H(Z) increases the high-frequency components of the sound signal to be transmitted, hence pre-compensating for the attenuation in these high-frequency components. After the pre-emphasis filtering, random noise can be effectively suppressed. We used a first-order high-pass filter [46] to implement pre-emphasis in signal pre-processing:

(1) $$H\left(Z\right)=1-\mu Z^{-1},$$

where the coefficient μ is typically within the range of (0.9, 1).

**Figure 2:**
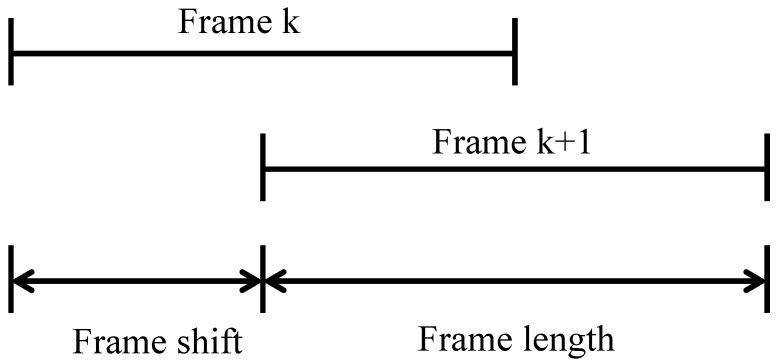
Relationship between frame shift and frame length.

The sound signal is a non-stationary signal [47] that usually remains stable between 50 and 200 ms. Therefore, features are extracted for frames whose frame size is within this range. To achieve a smooth transition between frames, a 50% overlap between consecutive frames is usually used in feature extraction. In our work, a frame is considered as a sample. The process of framing is to divide the pre-emphasized signal into multiple samples. Figure 2 shows the relationship between frame shift and frame length. A window function [48] is a function used to reduce signal interruption at the beginning and end of each frame. This is done by considering the next frame and integrating the frequency lines, thus making each frame smoothly interconnected.

The window function, which is mainly used to reduce spectral leakage and improve the fence effect [49], transforms the acquired signal block from a non-periodic signal to a periodic signal by weighting the time domain signal to meet the periodicity requirement of the Fourier transformation. As windowing is equivalent to convolution in the frequency domain of the measured signal, the result is equivalent to a weighted superposition of the window function spectrum after translating it to the original signal spectrum. As the original frequency domain signal along the window function frequency domain leaks out, the window function of the main flap width and side flap attenuation directly affects the performance of the window function.

**Figure 3:**
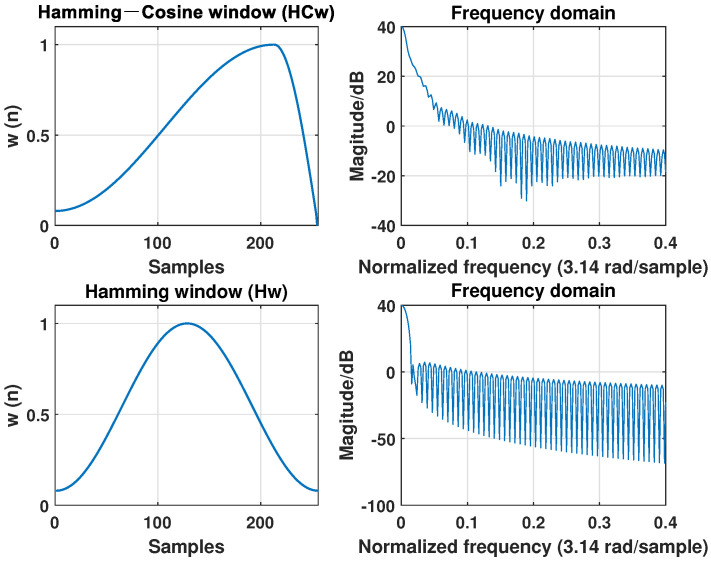
Comparison of Hamming and Hamming-cosine window.

The window function is characterized by its main lobe width and side lobe decay speed. The main lobe width and the attenuation of the side lobe affect the frequency resolution simultaneously: the smaller width of the main lobe and the faster the attenuation of the side lobe, the stronger the resolution of the frequency and the smaller the degree of leakage [50]. Therefore, to balance the tradeoff between the width of the main lobe and the width of the side lobe, as shown in Figure 3, we chose the Hamming-cosine window function [51], which has a wider main lobe than the Hamming window function, but a side lobe that decays faster.

(2) $$W\left(n\right)=\left\{\begin{array}{cc} 0.54-0.46\cos\left(\frac{2\pi n}{\frac{10}{6}N-1}\right), & n=0,\ldots ,\frac{5}{6}N-1\\ \cos\left(\frac{2\pi \left(n-\frac{5}{6}N\right)}{\frac{4}{6}N-1}\right), & n=\frac{5}{6}N,\ldots ,N-1 \end{array},\right.$$

where *N* denotes the number of samples in each frame.

Figure 3 shows a comparison of the Hamming window (Hw) and the Hamming-Cosine window (HCw) in the time and frequency domains. We observed that the main lobe of HCw is wider than that of Hw, but the side lobe is more attenuated. The experimental results [51] show that HCw has a better recognition performance.

### 4.2. Spatio-Temporal Feature Extraction

Feature extraction of sound signals is a key step in the fault diagnosis system and plays a decisive role in the classification performance. We extracted the temporal (time-dependent) and spatial visual features of sound signals using the FMFCC and spectrogram, respectively, as detailed below.

#### 4.2.1. Spatial Feature Extraction

The sensitivity of the human auditory system is unstable and varies with frequency. The Mel frequency domain describes the nonlinear properties of human ear frequencies [52], which can be represented by the following sound signal frequency relationship:

(3) $$f_{mel}=2595\log\left(1+f/700\right),$$

where $f_{mel}$ is the Mel scalar frequency and *f* is the frequency of the actual signal. We designed an MFCC-based feature extraction algorithm, which consists of several major steps.

Fast Fourier Transform (FFT): When the operating state of the transformer changes, the energy distribution of its sound signals in the frequency domain also changes. A fast Fourier transform is performed on the pre-processed signal. The obtained spectrograms are used as the extracted spatial visual feature:

(4) $$X\left(k\right)=\sum_{n=0}^{N-1}x\left(n\right)e^{-j2\pi nk/N},\;\left(0\leq k\leq N\right)$$

where X(k) is the spectrogram of the sound signals, x(n) is the windowed signal, and *N* is the number of sampling iterations of the Fourier transform.

#### 4.2.2. Temporal Feature Extraction

The steps commonly used to extract the MFCC include power spectrum calculation, the Mel triangle filter, the logarithmic spectrum, and the discrete cosine transform.

Power spectrum calculation: Taking the signal spectrogram X(k) as the square of its modulus, the power spectrum P(k) [53] is obtained as:

(5) $$P\left(k\right)=\frac{1}{N}{\left|X\left(k\right)\right|}^{2},$$

Mel triangle filter: The Mel spectrum is obtained from the triangular filter set of P(k). At each frequency, the product of P(k) and the filter $H_{m}\left(k\right)$ is calculated. M triangular filters are defined in the filter bank, which are linear in the Mel frequency coordinates. The span of each triangular filter in the filter bank corresponds to the Mel scale. The frequency response of the triangular filter $H_{m}\left(k\right)$ [54] is calculated as:

(6) $$H_{m}\left(k\right)=\left\{\begin{array}{cc} 0, & k<f(m-1),\\ \frac{k-f(m-1)}{f(m)-f(m-1)}, & f(m-1)\leq k\leq f(m),\\ \frac{f(m+1)-k}{f(m+1)-f(m)}, & f(m)\leq k\leq f(m+1),\\ 0, & k>f(m+1), \end{array}\right.$$

where *m* = 1, 2, …, 24, *k* = 1, 2, …, N/2−1, and f(m) is the center frequency. Here, we have

(7) $$\sum_{m=0}^{M-1}H_{m}\left(k\right)=1.$$

Logarithmic spectrum S(m): In order to make the results more robust to noise and estimation error, the logarithmic energy spectrum S(m) [9] of each frame is obtained by the logarithmic operation as:

(8) $$S\left(m\right)=\ln\left(\sum_{k=0}^{N-1}{\left|P(k)\right|}^{2}H_{m}\left(k\right)\right),\;\left(0\leq m\leq M\right),$$

where $H_{m}\left(k\right)$ is the filter bank, P(k) is the power spectrum, S(m) is the logarithmic spectrum, and *M* is the number of filter banks.

Discrete Cosine Transform (DCT): The DCT is performed on the above logarithmic spectrum to obtain the Mel frequency cepstrum coefficients C(n) [9]:

(9) $$C\left(n\right)=\sum_{m=0}^{N-1}S\left(m\right)\cos\left(\pi n\left(m-0.5\right)/M\right),n=1,2,\ldots ,L,$$

where *M* is the number of filter banks and *L* represents the order of the MFCC.

The MFCCs only reflect the static feature of sound signals. Since the human ear is more sensitive to the dynamic features of sound signals, the dynamic information of the sound spectrum also contains rich acoustic information, which can be used to improve the accuracy of the transformer fault diagnosis system.

We used the first-order difference (ΔMFCC) [9] of the MFCC and a relative feature (RMFCC) to represent the dynamic feature of sound signals, which reflects the changing tendency of the transformer operating state, calculated as:

(10) $$d\left(n\right)=\frac{1}{\sqrt{\sum_{i=-k}^{i=k}i^{2}}}\sum_{i=-k}^{i=k}i\cdot C\left(n+i\right),$$

(11) $$r\left(n\right)=\left\{\begin{array}{cc} C(n), & n<k,\\ \frac{C(n)-C(n-i)}{C(n+i)-C(n)}, & \;\mathrm{others}\;,\\ C(n)-C(n-1), & n⩾L-k, \end{array}\right.$$

where d(n) is the *n*th first-order difference, C(n+i) is a frame of acoustic parameters, d(n) is the first difference of the MFCC, and r(n) is the *n*th order relative MFCC feature. The value of *k* was set to 2 in our work.

The above feature parameters characterize different perspectives of sound signals from a power transformer. Considering the variation of the transformer’s operating state, we combined both static and dynamic features to describe the transformer’s sound signals. Directly superimposing the above features (36 dimensions) would increase the computational effort, as well as the number of dimensions of the feature parameters. Some parameters may contain less information, and some contain redundant information, which may affect the result of fault diagnosis if the contribution of these feature parameters is considered to be equal. Therefore, we should evaluate the degree of influence of each dimensional parameter on the recognition effect and select the parameters with the greatest influence on the recognition as the new feature parameters. Specifically, we combined the above static features and dynamic features together and obtained the contribution of each dimension by calculating the Fisher ratio of the feature dimensions.

The Fisher ratio [55] is calculated as:

(12) $$r_{\mathrm{Fisher}\;}=\frac{\sigma_{\mathrm{between}\;}}{\sigma_{\mathrm{within}\;}},$$

where $\sigma_{\mathrm{between}\;}$ is the interclass divergence matrix, which represents the sum of the interclass variances of the *k*th-dimensional component between various faults of the transformer, and $\sigma_{\mathrm{within}\;}$ is the intraclass divergence matrix, which represents the sum of the intraclass variances of the *k*th-dimensional component of a particular fault.

The interclass divergence $\sigma_{\mathrm{between}\;}$ [56] is defined as follows:

(13) $$\sigma_{\mathrm{Between}\;}=\sum_{j=1}^{M}{\left(u_{k}^{\left(j\right)}-u_{k}\right)}^{2},$$

where *M* is the number of transformer fault types, $u_{k}^{\left(j\right)}$ is the mean value of the *k*th-dimensional component of transformer fault *j*, and $u_{k}$ is the mean value of the *k*th-dimensional component of all faults.

The intraclass divergence $\sigma_{\mathrm{within}\;}$ [56] is defined as follows:

(14) $$\sigma_{\mathrm{Within}\;}=\sum_{j=1}^{M}\left[\frac{1}{n_{j}}\sum_{c\in w_{j}}{\left(c_{k}^{\left(j\right)}-u_{k}^{\left(j\right)}\right)}^{2}\right],$$

where $n_{j}$ is the number of samples of transformer fault *j* and $c_{k}^{\left(j\right)}$ is the *k*th-dimensional feature parameter of fault *j*.

**Figure 4:**
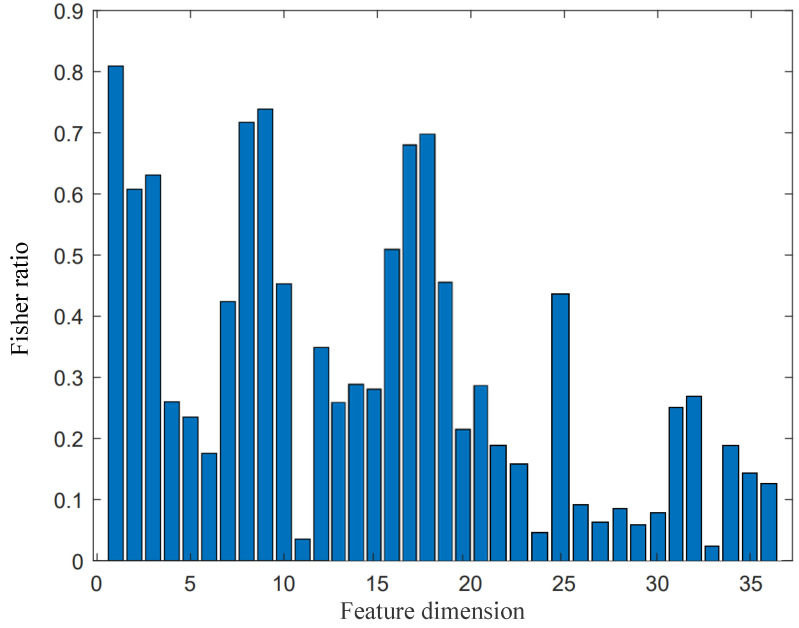
Fisher ratio of 36-dimensional parameters.

A larger Fisher ratio means that the feature parameters in this dimension contribute more to transformer fault diagnosis. We selected the composed new feature parameters for transformer fault diagnosis, referred to as Filtered-MFCC (FMFCC). As shown in Figure 4, we selected 1–4, 7–10, 12–19, 21, 25, 31, and 32 to form a new 20-dimensional parameter vector *V*:

(15) $$V=\left[S_{1},S_{2},S_{3}\ldots S_{31}\right].$$

The reduction of feature parameter dimensions not only removes the redundant information of feature parameters, but also mitigates the problem of the limited processing power and storage space of edge devices.

### 4.3. A Classifier Using a Parallel Dual-Layer, Dual-Channel Lightweight Neural Network

**Figure 5:**
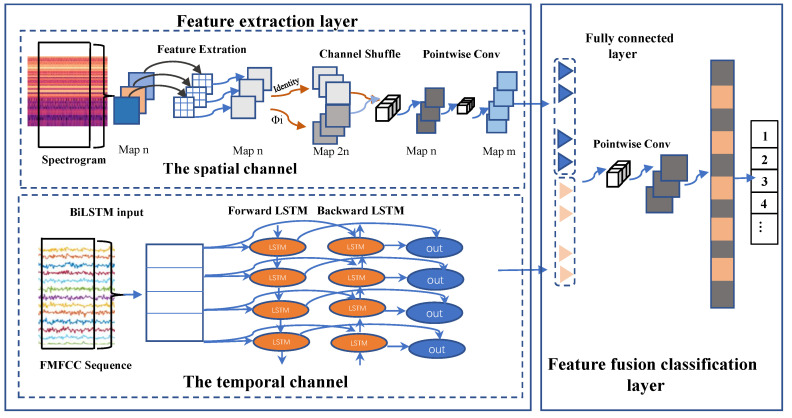
The network structure.

We extracted the temporal (time-dependent) and spatial visual features of sound signals using the FMFCC and spectrogram, respectively. To enable the quick identification of transformer fault types, as shown in Figure 5 we designed a parallel dual-layer, dual-channel lightweight neural network to achieve the fusion of sound signals in the spatial and temporal features. Compared with conventional networks, our proposed neural network has the advantages of fewer parameters and lower computational cost.

#### 4.3.1. Feature Extraction Layer

The feature extraction layer consists of the spatial channel and temporal channel, using the depth-separable convolution and recurrent neural-network-based Bidirectional Long Short-Term Memory (BiLSTM), respectively.

**Figure 6:**
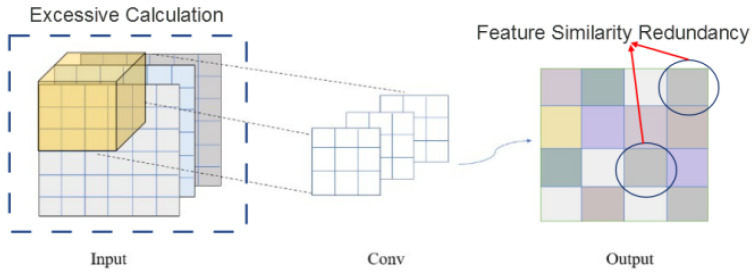
The general convolution.

Due to the limitation of the processing power of edge devices, we expect the feature extraction network to have a speedy inference and fewer computational operations. For this purpose, we designed a lightweight neural network for edge devices. For multi-channel (this channel is not the dual-channel network) inputs, most existing work employs general convolution (all-channel convolution). We performed convolutional feature extraction for each channel. Since there are three channels of input as shown in Figure 6, the feature extraction results in some redundant features, causing a waste of computational resources. Deep convolutional neural networks usually consist of a large number of convolutional operations, leading to more computational cost. At the convolution operator level, the general convolution has the inherent property of global spatial and channel feature extraction. The Depthwise-separable (DW) convolution completely separates spatial and channel feature extraction. The MobileNet [57,58] family has found the successful application of DW convolution and has recently made a number of improvements to reduce computational effort. ShuffleNet [59,60] restricts convolution operations to each group and performs channel shuffle, and it reduces the channel dimensionality by reducing the concatenation of computational effort.

In fact, the operation for generating 2n feature maps from any convolutional layer can be expressed as [61]:

(16) Y=X∗f+b,

where ∗ is the convolution operation, $X\in R^{n\times h\times w}$ is the input data (*n* denotes the number of input channels, and *h* and *w* are the height and width of *X*, respectively), and *b* is the bias term. As shown in Figure 6, the output feature maps of the convolutional layers often contain a large amount of redundancy. To reduce the redundancy of the convolution operations, we utilized the redundancy of the existing feature maps. As such, the redundancy is used to generate similar feature maps to obtain multi-channel (e.g., 2n feature channels) feature maps (feature maps of *n* feature channels obtained by some linear operations):

(17) $$Y^{\prime }=\Phi_{i}\left(Y_{i}\right),\forall i=1,\ldots ,n,$$

where $Y_{i}$ is the *i*th original feature map in *Y* and $\Phi_{i}$ is the *i*th linear operations.

With a linear mapping (Equation (17)), we relatively reduce a large number of operations while generating the same number of feature maps as the general convolutional layer. Next, we compare the computational process of linear mapping with that of general convolution. For example, we set the average kernel size for each linear operation to be d∗d and the convolution kernel size to be *k*. The format of the input data is defined as n∗h∗w, where *n* is the number of input channels, and *h* and *w* are the height and width of the input data, respectively. For comparison and the ease of deployment in the neural network, we set the size of the linear kernel to be the same as the size of the convolutional kernel.

The computational effort to obtain a 2n output channel using general convolution is:

(18) Com1=n×h×w×2n×k×k,

However, if 2n output channels are implemented using a linear mapping, the required computation is:

(19) Com2=n×h×w×n×k×k+n×h×w×d×d,

(20) $$\frac{Com2}{Com1}=\frac{n\times h\times w\times n\times k\times k+n\times h\times w\times d\times d}{n\times h\times w\times 2n\times k\times k}=\frac{n\times k\times k+d\times d}{2n\times k\times k}\approx \frac{n+1}{2n}\approx \frac{1}{2},$$

By comparing the computational effort for obtaining 2n output channels by general convolution and linear mapping, respectively, we conclude that our method reduces the computational effort by almost 50%.

The spatial channel uses the spectrogram as the input. Firstly, the input obtained by the spectrogram is generally convolved to enhance the dimensionality of the features in the process of extraction. Then, the high-dimensional features are convolved into a single channel, and each feature map corresponds to a convolution kernel for feature extraction. We generated the same number of feature mappings as the general convolutional layers by the aforementioned linear mapping to achieve further feature extraction phases with less computation compared with the general convolution.

However, there is no information exchange between the feature maps extracted from individual channels, which may generate feature barriers as the depth of the network increases. We added a channel shuffle layer to solve this problem, which assigns the features of different channels to the same group and performs feature extraction through a group convolution operation to facilitate feature exchange between different groups. Finally, we performed this pointwise on all extracted features to achieve feature dimensionality enhancement and further realized cross-channel information interaction. The features obtained by point convolution are the spectrogram features of the sound we extract. The complete neural network structure is shown in Figure 5.

The temporal channel extracts the FMFCC feature sequence of sound signals. LSTM [62,63] maps the output sequence or vector, and the hidden layer with self-circulating weights in LSTM enables the nodes in memory to preserve past information. Thus, LSTM can learn time sequence features by continuous input. Wang [64] and Giambattista [65] proposed the bi-directional LSTM-based sound event detection technique with better performance compared with DNN. Unlike acoustic events that occur in a short time, the duration of sound signals of transformer faults is longer. Therefore, we can improve the performance of classification by applying neural networks to sound signal fault classification.

Bi-directional LSTM (BiLSTM) is an improved LSTM with a bi-directional flow that processes sequences forward and backward and feeds them forward to the output layer. There are two hidden layers in BiLSTM, which compute the hidden sequence in both the forward and backward directions and update the output layer by the backward layer (from the last time step to the first) and forward layer (from the first time step to the last time step). We used the KMFCC feature sequence as the input to the temporal channel. The input sample dimension is 20, and each sample consists of 398 frames. The dropout of all layers has a probability of 80%.

#### 4.3.2. Feature Fusion Classification Layer

Now, we obtain the visual features (spectrograms) of the acoustic features extracted by the spatial channel and the FMFCC sequence features extracted by the temporal channel. We constructed dual-channels and formed a fully connected layer of 256 cells with 128 as each of their outputs.

Since the scale of both features is the same, we can directly fuse the two by the concat operation. In addition, in order to make further fusion between static and dynamic features and eliminate feature barriers, we used point convolution to perform feature extraction on the features after the concat operation. At the same time, since the dimension of the feature map is too large after performing the concat operation, which increases the excessive computational requirements, we used point convolution while also performing dimension reduction to reduce the subsequent computation. Finally, the data dimension is converted to 1 dimension by a fully connected layer. The output layer contains the same number of softmax nodes as the number of transformer fault types. The feature fusion classification layer is shown in Figure 5.

## 5. Experiments and Performance Evaluation

**Hardware.** We used a high-sensitivity sensor, Model HYCG-001, with a frequency response of 20 Hz to 20kHz. The signal-to-noise ratio and impedance are 65 dB (at 40 dB a meter) and 600–1000 Ω, respectively. In addition, the transducer is powered by a 12 V 1–2 A Direct Current (DC) power supply. We deployed the proposed LightFD system on a Raspberry Pi 4B device without relying on additional computing devices. The Raspberry Pi 4B used in our experiments is equipped with a Cortex-A72 CPU (1.5 GHZ ARMv8) with 8 G memory.

**Choice of edge computing platforms.** Edge devices are pervasive in our daily lives, as represented by smart watches and smart glasses, both of which are commercial devices that have become mature. In addition, they come with some boards for development and testing, which provide rich functions and are small in size for easy deployment. There are two types of development boards. One is mainly for AI development with hardware devices dedicated to computing, such as the GPU in NVIDIA TX2 and the VPU in Intel NCS2. This type of board can meet the requirement for complex AI development, but is also very expensive and does not represent a common computing edge device. The other is an ordinary development board, which meets most of the conditions and is relatively inexpensive for use in a wide range of applications. Such commonly used development boards include Raspberry Pi and Intel UP Squared. The CPU frequency of UP Squared is 2.5 GHz, while that of Raspberry Pi 4B is only 1.5 GHz, which is comparable in its category. Therefore, we believe that Raspberry Pi provides a suitable experimental platform for the deployment and testing of our system. Raspberry Pi 4B [66] was chosen for several reasons: (i) it is inexpensive and suitable for large-scale deployment in a variety of environments; (ii) its performance meets the requirements of an edge device and is representative of common types of edge devices.

**Figure 7:**
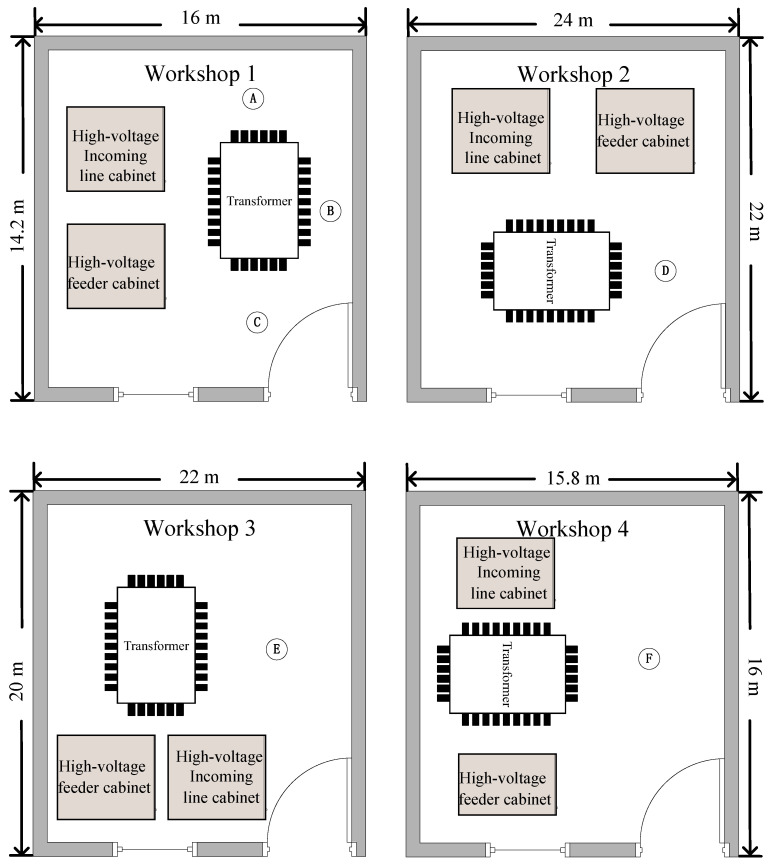
The schematic diagram of the transformer workshops. (A–F is the position of the sensor.)

**Data collection:** The data collection was conducted on a 110 kV three-phase dry-type transformer. Due to the wide variety of transformer faults, there is no public dataset available. Before conducting the experiment, we collected data in four workshops, whose schematic diagram is shown in Figure 7. We collected transformer faults for six common types of faults, whose specific descriptions are provided in Table 1. Note that both faults of “severe internal short circuit” and “internal breakdown short circuit” are internal short circuits, but there is a major difference in audibility. The sound of a severe internal short circuit is similar to the sound of boiling water. The sound of an internal breakdown short circuit is similar to a crackle sound and is usually caused by the moisture on the transformer and other factors creating a short circuit ring. In addition, a severe internal short circuit is transient, and its condition may lead to an internal breakdown short circuit, which is a permanent short circuit state, and hence is more critical and complex than a severe internal short circuit. We considered three locations of the sensors, A, B, and C in Workshop 1 in Figure 7. In addition, we placed sensors at positions D, E, and F in the other three workshops, respectively.

We performed experiments to evaluate the performance of fault diagnosis. The first experiment tests the overall diagnostic classification effectiveness of our proposed method. The second experiment shows the performance comparison with different feature extraction methods. Next, we compared the performance of the dual-channel network in our proposed classifier. Finally, we compared the effects of different sensor positions and numbers on the experimental performance.

To evaluate the proposed method, two measurements were used in each experiment: Precision and Recall, defined as:

(21) $$\;\mathrm{Precision}\;=\frac{TP}{TP+FP^{\prime }}$$

(22) $$\;\mathrm{Recall}\;=\frac{TP}{TP+FN^{\prime }}$$

where TP is the number of true positive results, TN is the number of true negative results, FP is the number of false positive results, and FN is the number of false negative results.

### 5.1. System Performance

**Figure 8:**
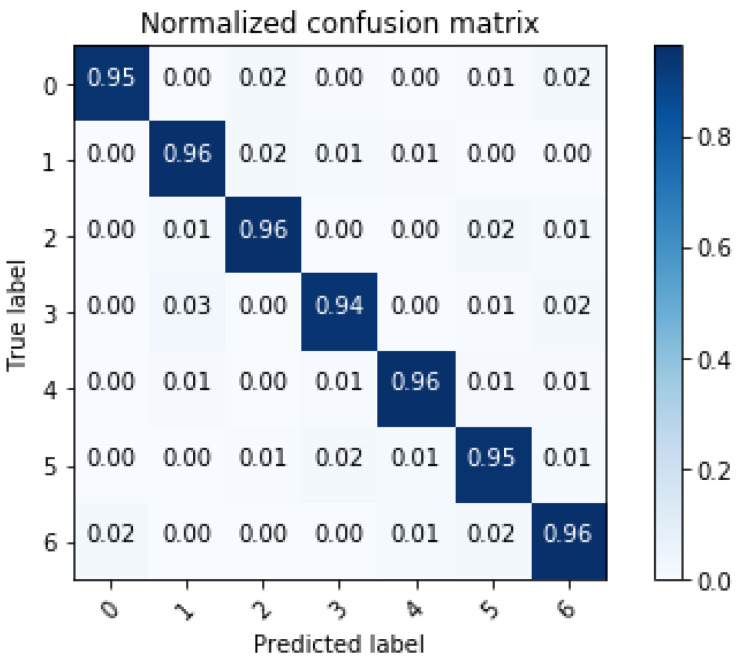
The confusion matrix of the related fault.

In order to present the detailed diagnostic classification results, as shown in Figure 8, we calculated the values of the confusion matrix by extracting the data under normal and six faults. We used the serial numbers in Table 1 to represent transformer fault types and 0 to indicate that the transformer works normally. Experimental results showed that our method achieves good performance in diagnostic classification. In particular, it performs well for faults such as internal short circuit (Serial Number 1) and severe internal short circuit (Serial Number 2), but the fault diagnostic effectiveness decreases in internal breakdown short circuit (Serial Number 3).

**Figure 9:**
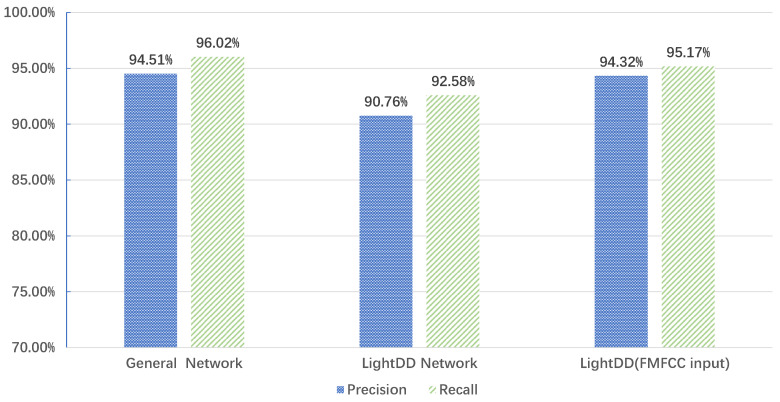
System accuracy in three cases.

We also evaluated the Precision and Recall of LightDD for general convolutional networks, LightDD, and FMFCC inputs. As shown in Figure 9, LightDD leads to lower Precision and Recall due to less convolutional operations and a simpler structure. However, we observed that when using the FMCC as the input of LightDD, the recognition Precision and Recall of the system can be up to 94.32% and 95.17%, respectively. This result is similar to that of a general network (with more convolutional operations). Therefore, we not only improved the recognition effectiveness of the neural network by enhancing the quality of the input features, but also ensured fast inference for edge devices with better recognition results.

### 5.2. Experimental on Feature Extraction for Sound Signals

**Figure 10:**
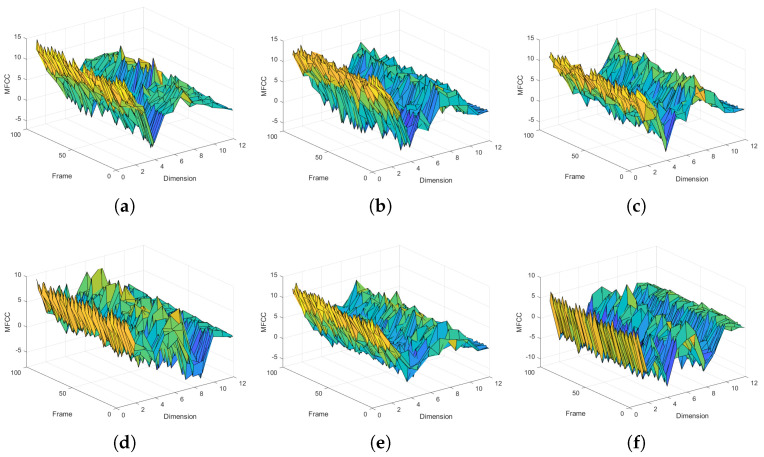
MFCC of transformer acoustic with different faults. (**a**) Large load start or internal short circuit. (**b**) Severe internal short circuit. (**c**) Internal breakdown short circuit. (**d**) Poorly grounded iron core. (**e**) Loose silicon steel or coil. (**f**) High voltage.

**Figure 11:**
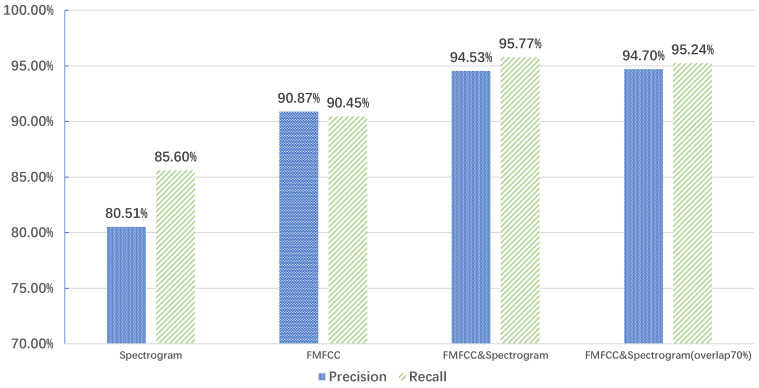
Comparison of different acoustic feature extraction methods.

In the spatio-temporal feature extraction, we extracted acoustic features including the acoustic spectrogram, MFCC, delta MFCC, and RMFCC. As shown in Figure 10, we extracted MFCC features for different faults. The experimental results showed that the MFCC feature profiles of the same fault type are similar. We calculated the delta MFCC and RMFCC based on the static MFCC with different faults. As shown in Figure 11, we compared the extracted spectrogram and FMFCC, respectively. The results showed that using the spectrogram had the lowest Precision and Recall, and the multidimensional spatio-temporal feature extraction method combining the spectrogram and FMFCC was more effective. In addition, we increased the overlap rate between frames to 70% and examined its effect on the diagnostic results before the extraction of acoustic features. As in the fourth case in Figure 11, we observed that the increase of the rate had almost no effect on the recognition. However, it would increase the number of frames divided by the sound signal, leading to an increase in computational effort.

### 5.3. Recognition Method and Computing Complexity Analysis

In this section, we compare the performance of the proposed classifier with the performance of the basic classification methods as shown in Table 2. The Precision and Recall of our fault diagnosis system are higher than the traditional machine learning method SVM.

In the transformer fault diagnosis system, we need to perform pre-processing, feature extraction, and construction and calculation of the convolutional recurrent neural network. The complexity of data pre-processing and feature extraction is almost negligible compared with the computational effort for constructing a convolutional recurrent neural network. Specifically, the proposed fault diagnosis method includes an offline training phase and an online fault diagnosis phase. The average time for the online fault diagnosis phase is 0.9 s. These results indicate that the computational power of the existing Raspberry Pi is sufficient to support our system.

### 5.4. Experiments with Different Locations and Numbers of Sensors

We compared the effects of different locations and different numbers of sensors on the diagnostic results using the two faults collected in Workshop 1. As shown in Figure 7, we collected the fault sound signals generated by the transformer at three sensor locations, A, B, and C, respectively, and also with three sensors present at the same time, as shown in the last column of Table 3. The results showed that the location and number of sensors have almost no effect on the diagnostic results. Therefore, in our experiments, we used one acoustic sensor and collected acoustic signals at Locations B, D, E, and F in different workshops, as shown Figure 7.

## 6. Conclusions

In this paper, we used non-contact sensors to collect sound signals of transformer faults and designed a fault diagnosis system based on a combination of the MFCC, spectrograms, and lightweight neural networks. The system was able to successfully detect and identify six types of transformer faults.

The following conclusions were drawn from our work:

- The extracted feature information reflects accurately the operating status of the transformer. An improved MFCC feature extraction method was proposed to characterize the dynamic features of acoustics. A multidimensional feature extraction method combining temporal and spatial features was proposed by combining the MFCC acoustic-based features with spectrograms.
- The proposed dual-layer, dual-channel neural network achieved satisfactory recognition performance and reduced computational effort by 50% compared to a generic convolutional network. This makes it possible to perform fast and high-precision recognition on low-end devices.
- Compared with the conventional SVM method, the designed fault diagnosis method improved the Precision and Recall rates by 4% and 1.6%, respectively.

## Figures and Tables

**Table 1:** Common transformer body sound anomaly analysis.

Anomaly	Fault Description and Causes	Number of Collected Signals	Serial Number
“Wawa”	Large load start-up or internal short circuit	1360	1
Sound of water boiling	Severe internal short circuit	1280	2
Crackle	Internal breakdown short circuit	1314	3
“Chichi”	Poorly grounded iron core	1250	4
“Jiji”	Loose silicon steel or coil	1370	5
“Wengweng”	High voltage	1154	6

**Table 2:** Performance comparison with SVM.

The Fault Serial Number	SVM		LightDD	
	Precision	Recall	Precision	Recall
1	90.12%	92.74%	94.95%	95.57%
2	87.41%	88.02%	94.95%	95.57%
3	92.47%	96.54%	95.76%	94.2%
4	93.30%	94.57%	96.99%	96.23%
5	91.85%	94.97%	94.42%	94.79%
6	87.77%	93.68%	90.78%	95.63%

**Table 3:** Performance comparison with different locations and numbers of sensors.

The Location of Sensors	Internal Breakdown Short Circuit		Loose Silicon Steel or Coil	
	Precision	Recall	Precision	Recall
A	95.65%	94.57%	94.13%	94.67%
B	95.87%	94.38%	94.38%	94.78%
C	95.78%	94.16%	94.39%	94.62%
A + B + C	95.7%	94.47%	94.41%	94.79%

## Data Availability

Not applicable.

## Abbreviations

The following abbreviations are used in this manuscript:

CNN	Convolution Neural Network
LightFD	Lightweight Fault Diagnosis
MFCC	Mel Frequency Cepstrum Coefficient
LPCC	Linear Predictive Cepstrum Coefficient
CFCC	Cochlear Filter Cepstral Coefficients
ANNs	Artificial Neural Networks
SVMs	Support Vector Machines
ΔMFCC	the first difference of MFCC
RMFCC	Relative-MFCC
FMFCC	Filtered-MFCC
LightDD	a parallel Dual-layer, Dual-channel Lightweight neural network
BiLSTM	Bidirectional Long Short-Term Memory
